# Performance Analysis of Time Synchronization Protocols in Wireless Sensor Networks

**DOI:** 10.3390/s19133020

**Published:** 2019-07-09

**Authors:** Linh-An Phan, Taejoon Kim, Taehong Kim, JaeSeang Lee, Jae-Hyun Ham

**Affiliations:** 1 School of Information and Communication Engineering, Chungbuk National University, Cheongju 28644, Korea; 2 The 2nd R&D Institute-Agency for Defense Development, Daejeon 34186, Korea

**Keywords:** time synchronization, clock, performance analysis, message delay jitter, wireless sensor networks

## Abstract

The time synchronization protocol is indispensable in various applications of wireless sensor networks, such as scheduling, monitoring, and tracking. Numerous protocols and algorithms have been proposed in recent decades, and many of them provide micro-scale resolutions. However, designing and implementing a time synchronization protocol in a practical wireless network is very challenging compared to implementation in a wired network; this is because its performance can be deteriorated significantly by many factors, including hardware quality, message delay jitter, ambient environment, and network topology. In this study, we measure the performance of the Flooding Time Synchronization Protocol (FTSP) and Gradient Time Synchronization Protocol (GTSP) in terms of practical network conditions, such as message delay jitter, synchronization period, network topology, and packet loss. This study provides insights into the operation and optimization of time synchronization protocols. In addition, the performance evaluation identifies that FTSP is highly affected by message delay jitter due to error accumulation over multi-hops. We demonstrate that the proposed extended version of the FTSP (E-FTSP) alleviates the effect of message delay jitter and enhances the overall performance of FTSP in terms of error, time, and other factors.

## 1. Introduction

Time (clock) synchronization is a crucial requirement for the operation of wireless sensor networks (WSNs). Various applications, such as time-division multiple access (TDMA) scheduling, device tracking, monitoring, and data fusion, require all nodes to have synchronized clocks. A significant number of studies regarding this problem have been conducted in recent years. Many protocols and algorithms have been proposed to achieve sub-microsecond accuracy in experimental test-beds [1,2,3,4,5,6,7,8,9,10,11,12,13,14,15,16,17,18,19,20,21,22,23]. However, unlike time synchronization in wired networks, wireless networks present additional challenges such as the uncertainty of wireless transmission, energy consumption, and mobility [24]. Moreover, the resource constraints on sensor devices (e.g., low computational memory and low data rate) must be considered when designing a time synchronization protocol. In fact, the performance of protocols can deteriorate due to many practical factors, such as hardware quality, message delay jitter, ambient environmental conditions, network topology, and protocol configurations. Therefore, it is necessary to understand the operation of time synchronization protocols and the possible effects of those factors before using them in a real network.

In this study, we conduct a performance analysis on the Flooding Time Synchronization Protocol (FTSP) [1] and Gradient Time Synchronization Protocol (GTSP) [11] which are representative time synchronization protocols with centralized and distributed mechanisms, respectively. Both protocols are listed in many surveys and comparative studies [25,26,27,28,29], and in particular, they are widely used as benchmarks for newly proposed time synchronization protocols [4,30]. Many protocols use similar methods to those of FTSP (least-squares regression) and GTSP (average consensus) in estimating clock drift. For example, [2,3,6,9] used least-squares regression as in FTSP, and [12,13,17,19,20,21] used the average consensus mechanism as in GTSP. In addition, the performance and stability of FTSP and GTSP have been proved by experimental testbed and simulation study [30,31]. Therefore, we believe that a performance study on FTSP and GTSP can provide comprehensive insights regarding how time synchronization protocols work in different scenarios as well as the advantages and disadvantages of each centralized and distributed mechanism.

The performance analysis is conducted to determine the diverse factors affecting the performance of time synchronization protocols, such as message delay jitter, time synchronization period, network topology, and packet loss ratio. In particular, FTSP is highly affected by message delay jitter and network size because message delay is accumulated as the number of hops from the reference node increases. To solve this problem, an extended version of FTSP (E-FTSP) is proposed and evaluated together with FTSP and GTSP from a diverse range of aspects. The main contributions of this study are as follows:

- A comprehensive study of the performance of time synchronization protocols under diverse factors is performed. The effects of these factors on FTSP and GTSP were analyzed to understand the behavior of time synchronization protocols. The simulation methods used can be applied to the evaluation of future protocols.
- We propose an enhancement of FTSP (E-FTSP) and evaluate its advantages. We explain the problem with FTSP caused by the accumulation of jitter and describe how our improvement minimizes it. The simulation results prove that E-FTSP improves upon the performance of FTSP significantly, especially in large-scale multi-hop networks.

The remainder of this paper is organized as follows. Related works are introduced in Section 2. In Section 3, we briefly review the time synchronization problem and the primary concepts behind FTSP and GTSP. Section 4 describes the simulation setup used for comparative analysis of the protocols. Section 5 discusses the evaluation results for FTSP and GTSP in terms of message delay jitter, length of synchronization period, topology, and packet loss. In this section, an extended version of FTSP (E-FTSP) is described and evaluated to prove its advantages over FTSP. Finally, we conclude the paper and suggest future works in Section 6.

## 2. Related Works

Evaluating the performance of time synchronization protocols in WSNs is a non-trivial task [32]. There are two common methods for achieving this task: using measurements on an experimental testbed or using a simulation tool. Testbed implementation can provide reliable, accurate, and practical results. However, the drawback of this method is that it is costly and time-consuming to build a large-scale testbed. Simulation tools provide great flexibility for validating and testing various scenarios. The accuracy of results from a simulation depends on how well the characteristics of the actual network are modeled. It is interesting to note that the researchers who proposed FTSP and GTSP used both aforementioned methods to demonstrate the precision of their proposed protocols. Despite that there has been a lack of diverse evaluations considering factors such as message jitter, network scale, and packet loss.

Several studies [26,27,28,29] have provided comparisons of time synchronization protocols based on theoretical analyses. However, they failed to provide quantitative performance analyses due to the absence of a simulation study. Other studies focused on classifying protocols into different categories. Essentially, synchronization protocols can be classified based on structure, synchronization approach, or message exchange mechanism [28].

Regarding structure-based classification, time synchronization protocols can be grouped into two categories: centralized and distributed time synchronization [33]. In centralized protocols [1,2,3,4,5,6,7,8,9,10], a reference node (also called a root or master node) will appear in the network. All other nodes in the network will synchronize their clocks to the reference node by receiving flooded messages from the reference node.

There are two approaches to flooding: slow flooding and rapid flooding. FTSP is slow flooding-based because each node waits a predetermined amount of time to propagate its time information. However, several studies [4,6,34] have pointed out that slow flooding decreases the accuracy of time synchronization protocols. Yildirim et al. [4] proposed Flooding with Clock Speed Agreement (FCSA) protocol to reduce the undesired effect of slow flooding. FCSA aims to force all nodes to run at the same speed by estimating the relative hardware clock rate of a reference node. To achieve this goal, the timing message of FCSA must carry additional information, such as hardware clock rate and compensated drift (rate multiplier).

In contrast, rapid flooding was employed in PulseSync [6] to prevent the problem of slow flooding. In PulseSync, nodes propagate the timing message from the reference node as fast as possible. However, rapid-flooding protocols must deal with the problem of collisions in the wireless network to achieve high performance. Moreover, this kind of flooding may not be possible in a low-duty cycle network because a node must wait for its transmission.

The recently proposed Adaptive Value Tracking Synchronization (AVTS) [5] protocol, uses the technique of adaptive value tracking to determine the rate of the reference clock and synchronize the entire network. Because AVTS does not require least-squares regression, it has a smaller memory footprint compared to FTSP, PulseSync, and FCSA. However, AVTS has slower convergence time compared to the least-squares-based protocols [5].

Another study [10] introduced three approaches to synchronizing the time in low-power wireless sensor networks. These approaches are self-correction, clock-prediction, and analytical-correction. The authors argued that high accuracy is not necessary for all WSN applications, and they aimed to provide a trade-off between synchronization accuracy and power consumption with a target accuracy of milliseconds.

Meanwhile, distributed time synchronization protocols [11,12,13,14,15,16,17,18,19,20,21,22,23] do not require any reference node. Therefore, they are robust to network topology changes and node failures. Most of them rely on consensus algorithms to coordinate independent clocks in the network. For example, the main idea of GTSP and Average TimeSynch (ATS) is to average local information repeatedly until all nodes eventually have a common clock. However, one drawback of this approach is its slow convergence speed [18]. To increase the convergence speed, the maximum time synchronization (MTS) [18] protocol was proposed. Essentially, MTS forces all nodes to follow the fastest clock in the network. Therefore, it can achieve faster convergence. However, clocks synchronized under MTS are faster than the desired clock. Moreover, if a malicious node with an abnormally high clock value enters the network, the synchronization may break down.

Besides the message passing-based time synchronization protocols, recent studies have leveraged existing infrastructures, such as Wi-Fi beacons [35] and electromagnetic energy radiation [36], to globally synchronize all nodes in the network. The advantage of these solutions is that the energy consumption of sensor nodes is reduced. However, the synchronization error of these protocols is higher than that of state-of-the-art solutions in WSNs (e.g., the mean synchronization error of [36] was 121 µs).

In summary, numerous time synchronization protocols in WSNs have been proposed in recent decades. However, there has not been a quantitative performance study on the diverse aspects affecting the performance of time synchronization in WSNs. The aim of this study is to provide comprehensive insights into the operations, problems, and possible improvements in time synchronization protocols through a quantitative analysis, which considers a diverse range of factors that affect performance, such as message delay jitter, time synchronization period, network topology, and packet loss ratio of a wireless link.

## 3. Time Synchronization in WSNs

### 3.1. Problem and Challenge

In a wireless network, each node has its own local clock, typically referred to as a hardware clock. This hardware clock’s timing is calculated by counting the pulses of an oscillator operating at a particular frequency. However, the output frequency of each oscillator varies according to the hardware age or ambient environmental conditions [5]. Consequently, each hardware clock is subject to varying clock drift. Because of this clock drift, even when two nodes exhibit the same initial time, they will exhibit different clock values after some time. The oscillators used in sensor hardware typically exhibit a drift from 30–100 ppm (parts per million) [11]. The hardware clock value $h_{i}\left(t\right)$ of node *i* can be modeled mathematically as follows:

(1) $$h_{i}\left(t\right)=\alpha_{i}t+\beta_{i}$$

where $\alpha_{i}$ is the hardware clock rate and $\beta_{i}$ is the initial hardware offset of node *i*. The difference between hardware clock rates is called the *clock skew*. Because the hardware clock operates continuously and should not be modified, a logical clock is defined to represent a global clock (synchronized clock). The value of the logical clock $L_{i}\left(t\right)$ for node *i* is calculated as follows:

(2) $$L_{i}\left(t\right)=\tau_{i}\left(\alpha_{i}t+\beta_{i}\right)+\delta_{i}=\tau_{i}\alpha_{i}t+\tau_{i}\beta_{i}+\delta_{i}$$

where $\tau_{i}\alpha_{i}$ is the logical clock rate and $\tau_{i}\beta_{i}+\delta_{i}$ is the logical clock offset of node *i*. The goal of the time synchronization protocol is to estimate $\tau_{i}$ and $\delta_{i}$ such that the logical times $L_{i}\left(t\right)$ of all nodes are equivalent.

Designing a time synchronization protocol for WSNs is very challenging. Most time synchronization approaches rely on message exchange between nodes. However, whenever a node generates a timestamp and sends it to other nodes for synchronization, the packet is subject to variations in delay before it reaches and is processed by receivers. Authors of FTSP analyzed the causes of delays and the magnitude of each in message transmissions [1]. Assuming that deterministic delays can be calculated exactly, the role of time synchronization protocols is to eliminate or reduce the effect of nondeterministic delays. Additionally, implementing existing protocols in real-world networks present particular practical challenges, such as packet loss, fast clock drifting, and mote limitations [37]. Therefore, the effects of these practical factors on the performance of time synchronization protocols should be analyzed and discussed carefully.

### 3.2. Flood Time Synchronization Protocol

FTSP [1] is one of the most well-known protocols for time synchronization in WSNs. In FTSP, a root node maintains the global time and synchronizes all other nodes in the network. FTSP uses MAC layer timestamps to eliminate most message delays and a linear regression table to compensate for clock drifts. To achieve a high accuracy, a special timestamp method is proposed to reduce the interrupt handling delay. By recording the timestamp at each byte boundary after SYNC bytes when it is transmitted or received, the timestamp precision can be improved. However, this mechanism is only available with calibrated hardware that uses byte-oriented radio chips (e.g., CC1000). Therefore, FTSP is not a purely software-based solution.

In FTSP, when a node receives sufficient timing messages (defined by the value of NUMENTRIES_LIMIT), it becomes a synchronized node and subsequently starts to forward the timing messages to other nodes. This design allows FTSP to synchronize in multi-hop networks. The root node is elected dynamically and can be re-elected in case of failure to ensure system robustness. The protocol does not build an initial tree; hence, it can adapt to dynamic topology changes. The experimental data indicate that the average synchronization error of FTSP is less than 3 µs per hop.

### 3.3. Gradient Time Synchronization Protocol

GTSP [11] is designed to optimize the synchronization error between neighboring nodes and is a completely distributed protocol that is based on the average consensus algorithm. Each node synchronizes with its neighboring nodes and no special reference node exists. Unfortunately, this property prevents GTSP from synchronizing to an external time source (e.g., UTC time) [16]. Similar to FTSP, GTSP uses a MAC layer timestamp technique and one-way message dissemination. GTSP avoids single points of failure and can dynamically adapt to topology changes. The researchers that proposed GTSP reported that the average synchronization error between neighboring nodes (4.0 µs) is slightly smaller than that of FTSP (5.3 µs), while the network synchronization error is higher.

## 4. Simulation Setup

The performance of time synchronization can vary even when implemented on the same hardware platform [32]. For example, with the same Berkeley motes platform, researchers that proposed Reference Broadcast Synchronization (RBS) [38] protocol reported 11 µs precision, while another study [39] reported 29 µs precision for RBS. It is difficult to conclude that the latter evaluation is incorrect because the difference in precision may have been caused by different conditions in the evaluation (e.g., network topology, message delays, clock drift). Therefore, to ensure fairness and repeatability in the comparison of time synchronization protocols, a simulation-based approach is more appropriate for this study.

**Figure 1:**
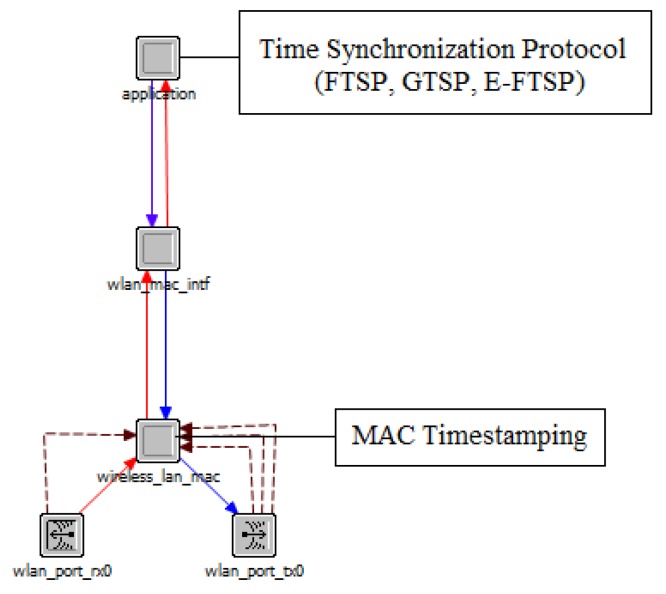
Node model in OPNET used to implement protocols in the evaluation.

The Riverbed Modeler (OPNET) [40] is used to evaluate the performance of FTSP and GTSP. The implementations of FTSP (https://github.com/tinyos/tinyos-main/tree/master/tos/lib/ftsp) and GTSP (https://github.com/phsommer/sinalgo-timesync) are referred from original implementation used to simulate these protocols. The MAC layer timestamp is implemented for both protocols. Figure 1 describes the node model in our simulation. A node model includes basic layers of a sensor node such as the application layer, MAC layer, and physical layer. The functions of time synchronization protocols are implemented at the application layer. However, the timestamp in the message is captured at the moment it is transmitted or received by the physical layer. Because GTSP does not require a reference node, it is unfair to compare its convergence time with that of FTSP which includes a leader election process. Hence, the first node (with ID = 1) is predefined as the reference node in FTSP so that the network can start the synchronization process immediately after the nodes are turned on. Each node in the network is set to a uniform random clock drift of ± 30–100 ppm. This clock drift range is reasonable because a sensor node typically uses inexpensive oscillators, and this is assumed in most studies [7,16,18,41].

The network size is 600 m × 600 m and the transmission range is set to 100 m, which allows distances of up to 12 hops in the network (grid topology 7 × 7) similarly to the evaluation of FTSP [1] and GTSP [11]. In a random topology, the positions of all nodes are uniformly and randomly distributed in the network. Noted that the following assumptions are made about the network: (1) the network is connected and (2) the connection link between nodes is symmetric. To support microsecond resolutions, the oscillator frequency is set to 1 MHz, implying that it generates one tick every microsecond. The settings of FTSP and GTSP are derived from the original studies of [1] and [11], respectively.

Each simulation execution is run for 2 hours (7200 seconds) and repeated 10 times. Different seeds are used in different executions to ensure that the random values are varied. However, it is important to note that in each particular simulation run, the simulation conditions (e.g., network topology, message delays, and clock drift) of each protocol are identical. The measurement data are collected after each synchronization round (30 s as default). Table 1 summarizes the default settings of the protocols and network, and a few settings are changed to evaluate the effects of different aspects on the protocols in each scenario. The simulation results are explained in the next section.

## 5. Evaluation Result

### 5.1. Effect of Message Delay Jitter

The term *message delay* is defined as the time elapsed from when a node starts transmitting a message until the receiver finally processes it. This amount of time is accumulated by all nondeterministic delays (e.g., channel access, interrupt handling, and propagation) and the deviation in deterministic delays. Message delay is inevitable in network communications. However, it is difficult to determine or estimate the total delay time exactly. Consequently, the message exchange process suffers from variations in delay, known as *message delay jitter*.

#### 5.1.1. Simulation Results

In this scenario, we wish to evaluate the effect of message delay jitter on the time synchronization protocols. This is a practical aspect of a real network, but it is typically ignored in a theoretical simulation [3,12,15,23,41]. Using the MAC layer timestamp, both FTSP and GTSP eliminated the send (receive) and channel access delays. However, a small delay caused by interrupt handling and message propagation remained. According to the experiment in [1], the likelihood delay can be several microseconds for interrupt handling. Therefore, a uniform random delay of up to 5 µs is set on every message sent.

**Figure 2:**
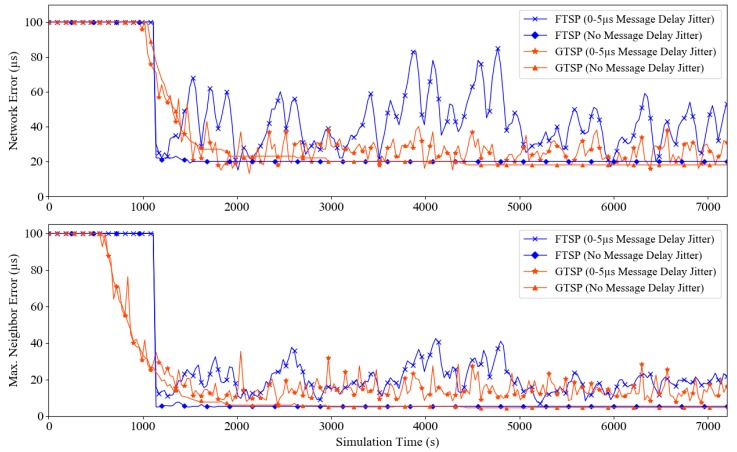
Network error and maximum neighbor error of FTSP and GTSP in two settings: without message delay and with delay jitter up to 5 µs. Random seed = 10.

**Figure 3:**
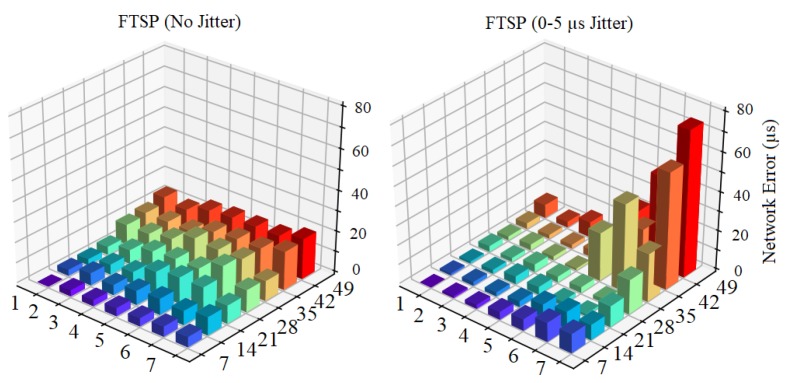
Synchronization error between reference node (at 1 × 1) and other nodes in grid topology in two settings: without message delay and with the message delay jitter up to 5 µs.

Figure 2 shows the synchronization error between FTSP and GTSP in two settings: with message delay jitter (up to 5 µs) and without (implying that the packet is received instantaneously). This is the representative result (with random seed = 10) from 10 independent simulation runs. From the figure, it can be seen that time synchronization error can be reduced significantly in an environment without message delay, and the maximum network errors of both FTSP and GTSP were only approximately 20 µs. Meanwhile, with message delay jitter, the maximum network errors of FTSP and GTSP increased significantly. However, the error scale of GTSP is smaller than that of FTSP. To clarify, Figure 3 shows the synchronization error of all nodes in a grid topology that used FTSP. These results demonstrate that a higher error occurs as the hop distance from the reference node increases in the presence of message delay jitter.

**Figure 4:**
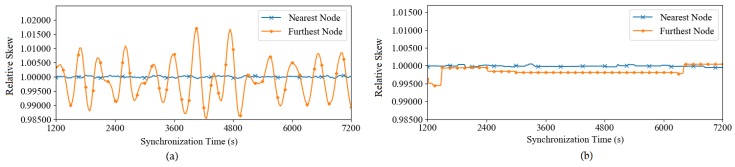
Relative skew of nearest node and farthest node with reference node in (**a**) FTSP and (**b**) E-FTSP with grid topology (7 × 7).

We analyze the effect of message delay on FTSP to obtain the cause and solution for this problem. We observed that the delay variation causes a fluctuation in the compensated drift. Figure 4a compares the logical skew of the nearest node with that of the farthest node with the reference node. Clearly, the logical skew must be close to 1 (implying nodes operating at the same speed as the reference node) and stable to maintain the precision of the synchronized time. In fact, even if the clock drift of the nodes is compensated completely, a minor offset error still appears at the receiving nodes because of the nondeterministic delay in message delivery. It is worth noting that the offset error in this case is not caused by insufficient compensation. Therefore, calculating the drift compensation again is not necessary. In FTSP, the nodes calculate and compensate the clock drift and clock offset each time they receive a timing message. However, these processes are independent of each other. Consequently, the nodes adjust their compensated drift unnecessarily because the offset is caused by delay variation and not by the difference in clock rate. Even though this adjustment of compensated drift is typically small, it is accumulated through each hop in a multi-hop network and will eventually become a large at the farthest nodes.

#### 5.1.2. Enhanced FTSP (E-FTSP)

To prevent unnecessary adjustment of the compensated drift, we propose a mechanism to control the drift compensation process. The primary concept is that the nodes will decide whether they must compensate for clock drift whenever they receive a timing message. To implement this concept, the procedure to handle a received message with a newly defined variable *estimatedDelay* is as below:

- If *offsetError* is smaller than *estimatedDelay*, the nodes will regard the previous compensated drift as sufficient and a recalculation will not be performed. In this case, the node must only compensate for the offset (see lines 3–5 in Algorithm 1).
- Meanwhile, if *offsetError* is larger than *estimatedDelay*, the nodes must calculate and compensate for the clock drift and clock offset using the algorithm of the original FTSP (see lines 7–9 in Algorithm 1).

This modification allows the nodes to skip updating the compensation of the clock drift if the offset error is trivial (smaller than *estimatedDelay*). Therefore, the nodes can reduce the skew fluctuation and computation overhead. Figure 4b shows that the difference between the relative skews of the nearest node and the farthest node with the reference node is insignificant after applying the proposed concept.

**Algorithm 1** Procedure to handle received message in E-FTSP
  1://Receive timing offset  2://Calculate offsetError  3:**if** (offsetError < estimatedDelay) **then**  4: //Compensate for offset only  5: compensate_offset(offsetError);  6:
**else**
  7: //Compensate for offset and drift  8: compensate_offset(offsetError);  9: compensate_drift();10:
**end if**



**Figure 5:**
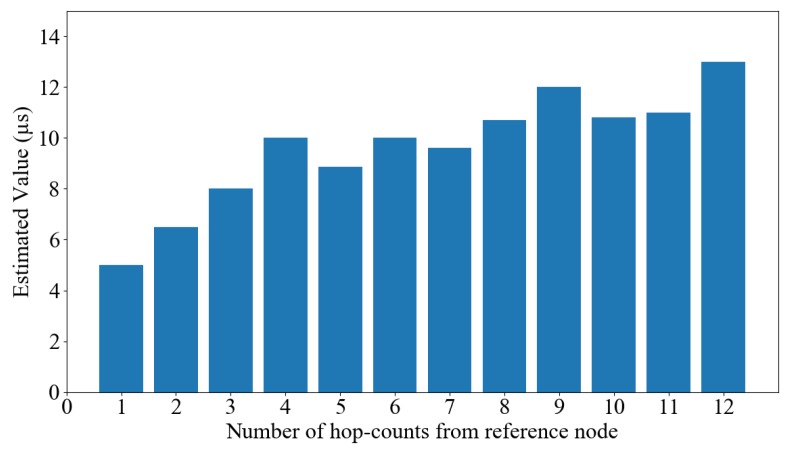
Average *estimatedDelay* value of nodes at same hop distance to the reference node.

To calculate *estimatedDelay*, we first analyze how FTSP stores the timing message in the regression table. Table 2 shows the regression table, where the offset error of node *i* is calculated as:

(3) $$O_{i}\left(t\right)=L_{j}\left(t\right)-h_{i}\left(t\right)$$

where $L_{j}\left(t\right)$ is the global clock value included in the message from node *j*, and $h_{i}\left(t\right)$ is the hardware clock value of node *i*. Assuming that the clock drifts of nodes *i* and *j* remain constant in the short-term (several messages), the difference in hardware clock $h_{i}\left(t_{n}\right)-h_{i}\left(t_{n-1}\right)$ should be constant if the messages are sent periodically. Subsequently, the difference between two continuous messages $O_{i}\left(t_{n}\right)-O_{i}\left(t_{n-1}\right)$ should theoretically be constant. However, because of message delay jitter, the value of $O_{i}\left(t_{n}\right)-O_{i}\left(t_{n-1}\right)$ varies. Therefore, (3) becomes

(4) $$O_{i}\left(t\right)=L_{j}\left(t\right)-h_{i}\left(t\right)+\sigma \left(t\right)$$

where σ(t) is a random delay in message delivery. Although the value of σ(t) varies each time the message is sent, the maximum value of σ(t) can be calculated as

(5) $$max_{\sigma }=\frac{max\left(O\left(t_{n}\right)-O\left(t_{n-1}\right):n\in \left\{1\ldots N\right\}\right)}{2}$$

where N is the number of entries in the regression table and $max_{\sigma }$ is the *estimatedDelay* value. Figure 5 shows the average *estimatedDelay* value of nodes that have the same hop distance to the reference node. The maximum delay in message delivery is 5 µs. The proposed protocol can estimate the maximum delay exactly with nodes that are received directly from the reference node (hop 1), and *estimatedDelay* slightly increases with further hops.

**Figure 6:**
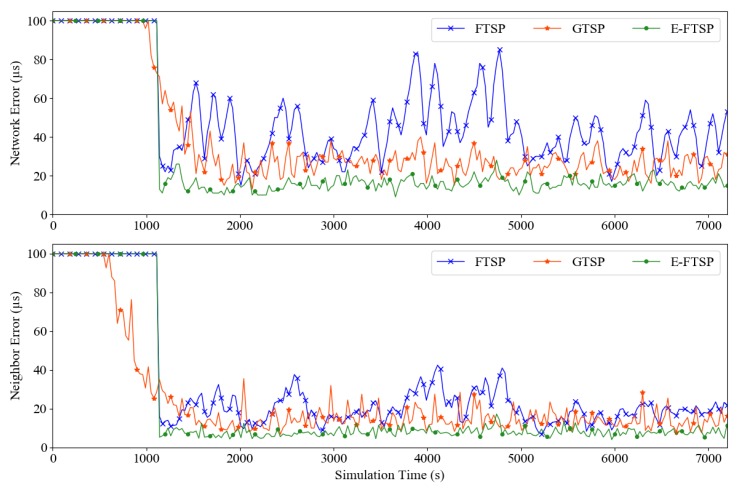
Network errors and maximum neighbor errors of FTSP, GTSP, and E-FTSP in the presence of message delay jitter.

In addition to calculating *estimatedDelay* automatically, it is possible to predefine the value of this variable. In a homogeneous WSN, the maximum and average delays can be obtained through experiments and manually configured in the software. This allows for the complexity of the algorithm to be reduced while maintaining the efficiency of the proposed protocol. We continue the first scenario by comparing the performances of E-FTSP, FTSP, and GTSP in the presence of message delay jitter. Figure 6 indicates that E-FTSP reduced the synchronization error significantly compared to FTSP. The maximum network synchronization error of the E-FTSP is only approximately 20 µs and the maximum neighbor synchronization error is approximately 10 µs. These synchronization errors are equivalent to the results for FTSP and GTSP in a no-delay environment. However, it is noteworthy that E-FTSP exhibits the same convergence time as FTSP. In addition, E-FTSP exhibits a smaller synchronization error than GTSP under the same conditions.

### 5.2. Effect of Synchronization Period

**Figure 7:**
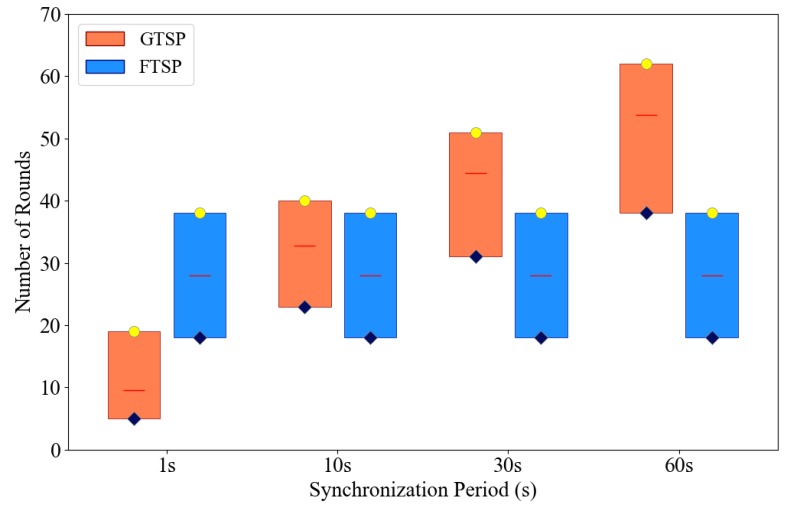
Number of rounds required to synchronize entire network in FTSP and GTSP with different synchronization periods.

Choosing a proper synchronization period when implementing a time synchronization protocol is a trade-off problem. A short synchronization period allows a network to become synchronized quickly, whereas a long period allows the nodes to save energy. In this simulation scenario, the effect of the synchronization period of each protocol is analyzed from the perspective of synchronization speed (convergence time) and accuracy. We consider only the number of synchronization rounds instead of the amount of time for comparison. It is apparent from Figure 7 that the number of rounds required to synchronize the entire network is lower for a shorter period with GTSP, while that in FTSP tends to be constant regardless of the duration of the synchronization period. The reason for this phenomenon is that GTSP gradually adjusts the clock rate of each node to a common clock rate. A “common clock rate” implies that the difference in clock rate between nodes must be trivial such that the offset will be small after a synchronization period. However, with a short synchronization period, the logical clock values are synchronized regularly even though the clock rate of each node is not strictly common. In other words, GTSP does not require many rounds to achieve a common clock rate in a short synchronization period.

**Figure 8:**
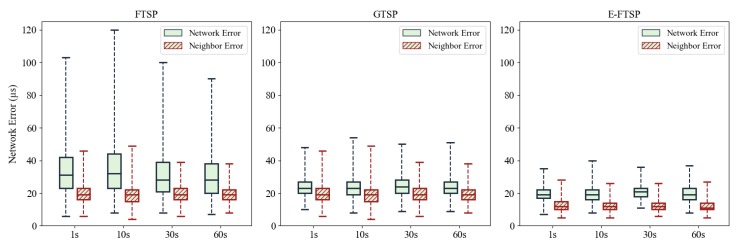
Network error and maximum neighbor error of FTSP, GTSP and E-FTSP with different synchronization periods.

Regarding the synchronization error, it is interesting that the short period does not improve the accuracy of both protocols but slightly increases the error in FTSP as shown in Figure 8. The simulation is repeated 10 times, and 300 points of data were collected after the network had achieved synchronization. The boxplots describe the distribution of the maximum network error and maximum neighbor error for each protocol. In general, changing the synchronization period does not increases the synchronization error of GTSP or E-FTSP. However, FTSP exhibits a higher synchronization error when it sends a message in over short period. As explained previously, FTSP incurs clock skew fluctuations due to message delay jitter. This problem is even more serious in a short synchronization period. Meanwhile, E-FTSP always demonstrates outstanding performance; its synchronization error is smaller than those of FTSP and GTSP for any synchronization period.

### 5.3. Effect of Topology

Topology is an important factor that affects both the time synchronization protocols and other protocols in the network (e.g., routing protocol). The researchers that proposed FTSP and GTSP claimed that both protocols operate well in different topologies. In this scenario, we wish to demonstrate how different topologies affect the performance of FTSP and GTSP.

#### 5.3.1. Position of Reference Node in FTSP

**Figure 9:**
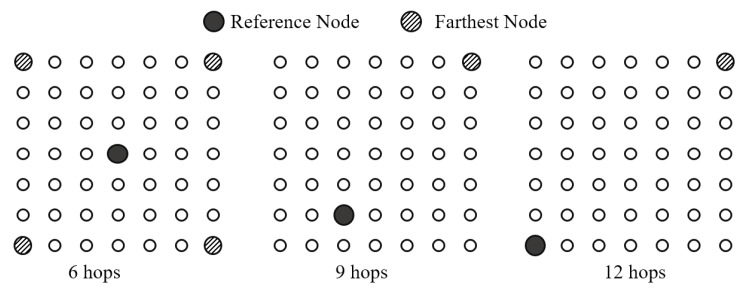
Hop distance between reference node (root) and farthest node in grid topology.

**Figure 10:**
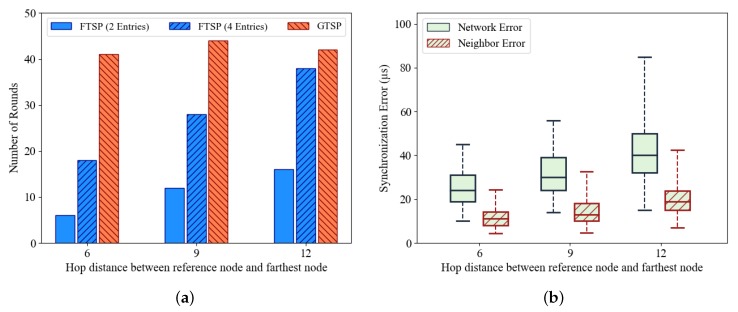
(**a**) Number or rounds required to synchronize entire network with different hop distances between reference node and farthest node. (**b**) Synchronization error of FTSP with different hop distances between reference node and farthest node.

FTSP in a multi-hop network has been reported to have poor performance. To understand the effect of hop distance from the reference node, the maximum hop distance between the reference node and the farthest node(s) is changed, as shown in Figure 9. The results of this simulation are shown in Figure 10. It is clear that the number of rounds required to synchronize the entire network in FTSP depends on the hop distance to the farthest node(s) as shown in Figure 10a. Because GTSP does not require a reference node, it is not affected by this. To reduce the waiting time in the “flooding” message, the NUMENTRIES_LIMIT value in FTSP setting can be reduced (e.g., two entries). This configuration allows the nodes to start forwarding the timing message after receiving a sufficient number of messages. The position of the reference node affects the network synchronization error as shown in Figure 10b. Thus, the choice of reference node is an important factor in achieving better performance with FTSP. In a static topology, it is easy to choose a reference node that allows the hop distance values to be minimized. However, in a dynamic network, the position of the reference node can be changed regularly. Additionally, FTSP does not provide any mechanism to ensure that a center node will become a leader in the election process. Hence, this is an open issue with FTSP.

#### 5.3.2. Distribution of Nodes in GTSP

**Figure 11:**
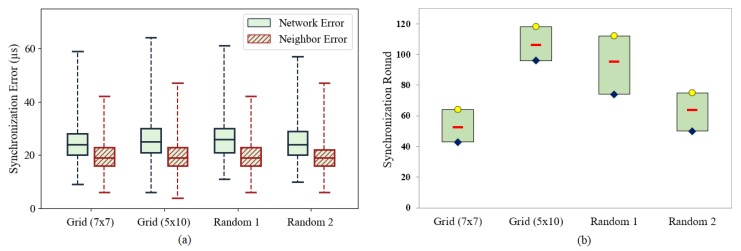
(**a**) Network error and maximum neighbor error of GTSP with different topologies. (**b**) Number of rounds required to synchronize entire network in GTSP with different topologies.

Because GTSP is not affected by the position of the reference node, this subsection extends the previous simulation of GTSP to different topologies. We do not include FTSP in this scenario because the convergence of FTSP depends on the hop distance from the reference node, as demonstrated in the previous evaluation. Figure 11b shows that the number of rounds required to synchronize the entire network differs according to the network topology. Even with the same topology, the number of rounds also varies in each execution. With each topology, the simulation is repeated 15 times with different seeds. Consequently, the initial clock drift and the time to broadcast the message of each node are different in every simulation. Therefore, the synchronization speed and the agreement clock rate also vary. However, Figure 11a shows that after achieving synchronized status, the synchronization error is similar among the various topologies.

**Figure 12:**
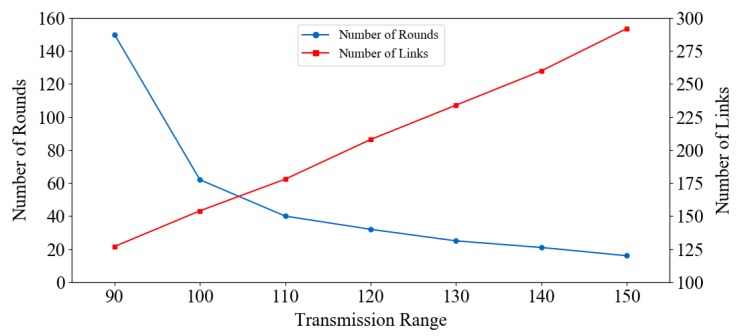
Number of rounds required to synchronize entire network in GTSP with different transmission ranges.

Li [42] demonstrated that convergence speed depends on the distribution property of a network. In detail, it depends on the number of neighbors (links) of each node. Hence, increasing the density and node connections in a network will increase the convergence speed of GTSP. A simple method to increase the convergence speed is to increase the transmission range of the nodes, as shown in Figure 12. In this scenario, the transmission range of nodes in a random topology (50 nodes) is increased gradually. As a result, the number of links is increased, and the convergence time is reduced. Although this is not always possible in WSNs because the transmission range is limited by hardware. However, it can provide insights into the acceleration of convergence time by exploiting the number of links in GTSP [43].

#### 5.3.3. Large-Scale Network

**Figure 13:**
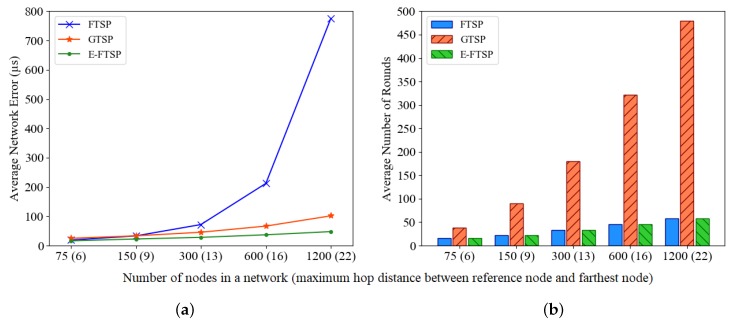
(**a**) Average network error of FTSP, GTSP, and E-FTSP according to the network size (number of nodes). (**b**) Average number of rounds required to synchronize entire network of FTSP, GTSP, and E-FTSP according to the network size (number of nodes).

In this subsection, we analyze the performance of FTSP, GTSP, and E-FTSP in term of network scalability, which is an important criterion for network protocols. Network scalability can be defined as the overall network performance may not degrade regardless of the network size and the number of nodes. Since the effect of the network density is discussed in Section 5.3.2, this subsection focuses on the effect of the network size, especially the number of nodes, by fixing network density for all experiments. A random topology was used as same as previous evaluations, but the number of nodes is increased from 75 to 1200 nodes as a logarithmic scale. Please note that the network size is also enlarged to maintain the same network density for each experiment. As explained in Section 5.3.1, the synchronization errors of FTSP are accumulated through multi-hops from the reference node. Therefore, we marked the number of nodes together with the maximum hop distance between the reference node (located at the center of the network) and the farthest node in x-axis of Figure 13.

Figure 13a shows that network synchronization error of FTSP grows exponentially with network size (hop distance), whereas that of GTSP only slightly increases. This indicates that GTSP is not affected by hop distance due to its inherent design of the distributed algorithm. Remarkably, the synchronization error of E-FTSP tends to be constant with increasing network size. This proves that E-FTSP minimizes the effect of error accumulation through multi-hops.

Figure 13b describes the average number of rounds required to complete synchronization of each network. It indicates that to achieve less than 100 µs accuracy the convergence time of GTSP increases proportionally to the network size, while those of FTSP and E-FTSP only slightly increase. It proves that the convergence time of GTSP highly depends on the maximum hop distance of a network. In contrast, the convergence times of FTSP and E-FTSP only depend on the hop distance between the reference node and the farthest node. Because the reference node is located at the center of the network, both FTSP and E-FTSP have tendencies to increase in convergence time as network size increases, but the impact is relatively small compared to that of GTSP.

In summary, FTSP has tendency that time synchronization error increases for the large-scale network while GTSP requires long convergence time proportionally to the network size. Therefore, neither are suitable for operating in large-scale networks. In contrast, the simulation results prove that E-FTSP provides high accuracy and fast synchronization regardless of network scale.

### 5.4. Effect of Packet Loss

**Figure 14:**
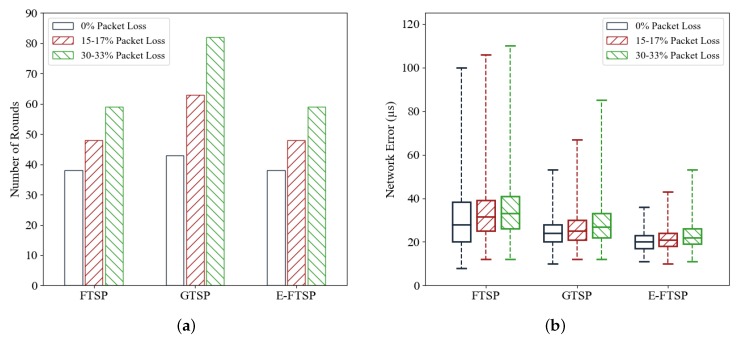
(**a**) Number or rounds required to synchronize entire network for different packet loss ratios. (**b**) Network synchronization error of each protocol with different packet loss ratios.

Packet loss might occur frequently in a practical network, especially in wireless networks. Packet loss can be caused by many factors, such as collision during transmission, signal quality, hardware, and software issues [44]. In this simulation scenario, we do not focus on the cause of packet loss in WSNs. Instead, the performance of each protocol is evaluated for different packet loss ratios. It is predicted that the packet loss will deteriorate the performance of the time synchronization protocols. Figure 14a shows that the number of rounds (convergence time) increases gradually with the packet loss ratio. Similarly, the network synchronization error increases with the packet loss ratio, as shown in Figure 14b. Generally, the network synchronization error of E-FTSP is still smaller than those of GTSP and FTSP under the same conditions. It is noteworthy that the network still achieves synchronization even in high-error environments such as one with 30% packet loss. This is because the time synchronization protocols send messages periodically; hence, it is acceptable if packets are lost in some synchronization rounds. In other words, packet loss does not cause abnormal errors in E-FTSP (and in FTSP and GTSP). This proves the robustness of these protocols.

## 6. Conclusions

In this paper, we presented a performance analysis of time synchronization protocols under the effects of different factors, such as message delay jitter, synchronization period, network topology, and packet loss. From the simulation, our conclusions are as follows:

- Message delay jitter can be considered to be the primary factor affecting the performance of time synchronization protocols. In particular, it causes fluctuations in the clock skew through multi-hop flooding and reduces the accuracy of FTSP significantly. An extended version of FTSP (E-FTSP) was proposed to reduce the effect of message delay jitter and it demonstrated outstanding performance compared to FTSP and GTSP, especially in a large-scale network.
- Regarding network topology, the position of the reference node affects the convergence time and synchronization error of FTSP and E-FTSP. In detail, the hop distance from the farthest node(s) should be as small as possible to achieve a high performance in FTSP and E-FTSP. Meanwhile, the distribution of nodes, especially the number of links between nodes in the network, affects the convergence time of GTSP. In detail, the convergence speed of GTSP increases with the number of links. In a small-scale network, there is no significant difference between the aforementioned protocols in term of synchronization error and convergence time. However, in a large-scale multi-hop network, FTSP has huge synchronization error, and GTSP has very slow convergence time. Meanwhile, E-FTSP provides more accurate and faster time synchronization regardless of network scale.
- Changing the synchronization period (interval) does not reduce the synchronization errors of FTSP and GTSP. A short synchronization period slightly increases the synchronization error in FTSP. Interestingly, a short synchronization period reduces the number of rounds required to achieve convergence in GTSP. Time synchronization protocols require short intervals for fast synchronization and long intervals to save energy. Therefore, adaptive synchronization protocols should be further investigated.
- Packet loss clearly increases the convergence times and the synchronization errors of FTSP and GTSP. However, the network still achieves a synchronized status even when approximately one third of packets are lost. This proves the robustness of FTSP, GTSP, and E-FTSP.

Apart from accuracy, convergence speed, robustness, and scalability, other attributes of a time synchronization protocol, such as energy efficiency and time complexity of algorithms, also need to be evaluated before implementing them in a real system. Thus, it is expected that studies on these attributes will improve the feasibility of real systems.

In our simulation, we did not evaluate the performance of FTSP and GTSP is mobile and the fast drifting environments. The mobility of the nodes in an ad-hoc network may generate unexpected results and problems. Although it has been claimed that FTSP and GTSP can operate well under dynamic topology changes, it is important to evaluate the performance of time synchronization protocols in a dynamic environment. These evaluations will be performed in future works. We believe that this study furthers our understanding of the performance of time synchronization protocols in real networks. Moreover, this work provides insights into the optimization of time synchronization protocols. 

## Figures and Tables

**Table 1:** Setting values in simulation.

Category	Setting	Value
	Topology	Grid/Random
	Number of Nodes	50
	Transmission Range	100 m
	Network Coverage	600 m × 600 m
Common	Initial Clock Drift	±30–100 ppm
	Synchronization Period	30 s
	Oscillator Frequency	1 MHz
	Simulation Time	7200 s
	Number of Executions (per scenario)	10
	NUMENTRIES_LIMIT	4
FTSP	Initial Root Node ID	1
	Regression Table Size	8
GTSP	JUMP_THRESHOLD	10 µs

**Table 2:** Regression table of a node.

Index	Local Time	Offset
1	h($t_{1}$)	O($t_{1}$)
2	h($t_{2}$)	O($t_{2}$)
3	h($t_{3}$)	O($t_{3}$)
4	h($t_{4}$)	O($t_{4}$)
5	h($t_{5}$)	O($t_{5}$)
6	h($t_{6}$)	O($t_{6}$)
7	h($t_{7}$)	O($t_{7}$)
8	h($t_{8}$)	O($t_{8}$)
